# A Capacitance-To-Digital Converter for MEMS Sensors for Smart Applications

**DOI:** 10.3390/s17061312

**Published:** 2017-06-07

**Authors:** Javier Pérez Sanjurjo, Enrique Prefasi, Cesare Buffa, Richard Gaggl

**Affiliations:** 1 Electronic Technology Department, Carlos III University of Madrid, Madrid 28911, Spain; eprefasi@ing.uc3m.es; 2 Infineon Technologies, RF & Sensors, Villach 9500, Austria; Cesare.Buffa@infineon.com (C.B.); richard.gaggl@infineon.com (R.G.)

**Keywords:** MEMS, CDC, pressure sensor, capacitive sensors, dual-slope, low power

## Abstract

The use of MEMS sensors has been increasing in recent years. To cover all the applications, many different readout circuits are needed. To reduce the cost and time to market, a generic capacitance-to-digital converter (CDC) seems to be the logical next step. This work presents a configurable CDC designed for capacitive MEMS sensors. The sensor is built with a bridge of MEMS, where some of them function with pressure. Then, the capacitive to digital conversion is realized using two steps. First, a switched-capacitor (SC) preamplifier is used to make the capacitive to voltage (C-V) conversion. Second, a self-oscillated noise-shaping integrating dual-slope (DS) converter is used to digitize this magnitude. The proposed converter uses time instead of amplitude resolution to generate a multibit digital output stream. In addition it performs noise shaping of the quantization error to reduce measurement time. This article shows the effectiveness of this method by measurements performed on a prototype, designed and fabricated using standard 0.13 µm CMOS technology. Experimental measurements show that the CDC achieves a resolution of 17 bits, with an effective area of 0.317 mm^2^, which means a pressure resolution of 1 Pa, while consuming 146 µA from a 1.5 V power supply.

## 1. Introduction

In recent years, due to the Internet of Things (IoT)-related market, many new applications have been introduced in the microelectronics field. In this new scenario, sensors and their readout circuits are becoming essential. A new generation of sensors, integrated Microelectromechanical Systems (MEMS), is providing the required features and functionalities, and therefore, nowadays they represent a good segment in this market.

Integrated MEMS capacitive sensors are widely used for measuring different physical quantities as they benefit from their small size and integration in miniaturized Inertial Measurement Units (IMUs). Indeed the current market trend is to integrate 10 axes in a single chip: accelerometers and gyros are mature, several solutions have been recently investigated for the three magnetic axes and research is ongoing for pressure [1].

Due to the big amount of applications, many different topologies are needed. In order to reduce the cost of development, the area and time to market, a generic sensor interface seems to be the logical next step in the sensor market. The design of a Capacitance-to-Digital Converter (CDC), that can be connected to the output of any capacitive MEMS and which converts a physical value into a digital value, is of interest for most products. Topologies that can be adapted to different applications by digital configuration without losing performance are becoming more important.

The typical approach for high resolution CDCs is the use of charge transfer between capacitors to convert the sampled capacitance to a voltage which is then used as input for a high order multi-bit switch-capacitor (SC) Sigma-Delta ADC which provides high resolution with large area and power demanding blocks [2,3,4]. In parallel to these solutions different approaches (like period modulation or delay-chain discharge) try to reduce area and power consumption [5,6]. However, keeping the high resolution which is present in the first solution based on multi-bit SC-ΔΣ ADCs with these alternative solutions is still a challenge.

This paper presents an extension of the work reported in [7], showing measurements from a fabricated prototype. In order to be area and energy efficient the proposed architecture is based on the noise-shaping integrating dual-slope (DS) topology of [8,9,10]. Contrary to standard multi-bit ΔΣ ADCs, this solution uses time instead of amplitude resolution and therefore, the same performance is achieved but with single bit circuitry. 

The main strengths of the proposed CDC are: (1) intrinsically small sensitivity to temperature and process variations; (2) simplicity of trimming offset and gain to correct the sensor parameter spread; and (3) area and energy efficient implementation compared to traditional approaches, while maintaining the performance. 

This paper is organized as follows: Section 2 focuses on MEMS physics, the transduction principle to measure the pressure; the proposed architecture at system level is explained providing a time diagram for the synchronization of blocks. The readout circuit at the system level with some circuit design details are shown in Section 3. Section 4 describes the experiments done on the prototype and reports the achieved results. Finally, Section 5 presents a discussion where this solution is compared with the state-of-the-art and our conclusions.

## 2. MEMS Topology

**Figure 1:**
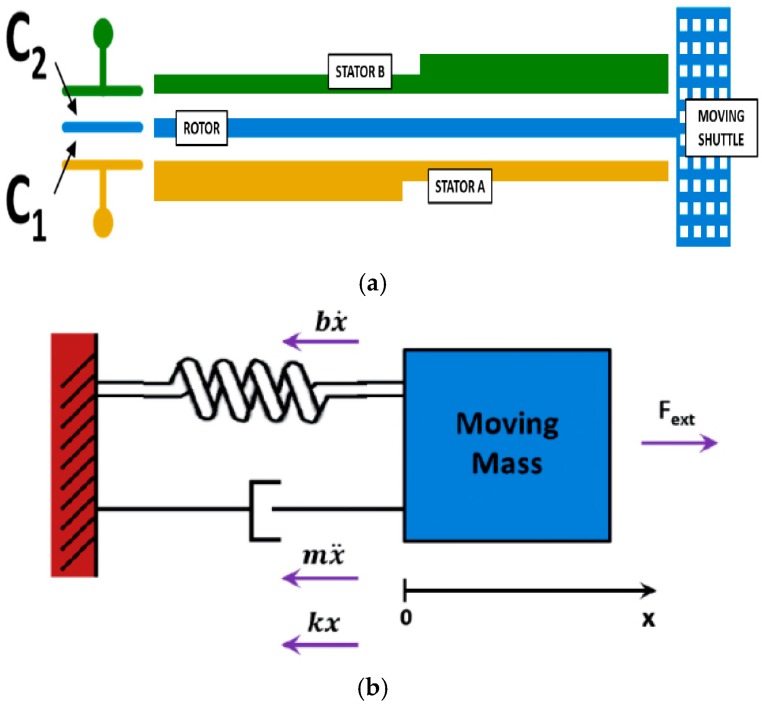
(**a**) Sketch of a typical differential capacitive sensing cell for a MEMS structure. Stators A and B are anchored to the chip substrate and they form a differential capacitor with rotor; (**b**) Diagram with forcing acting on a suspended mass.

MEMS are microscale systems made of both mechanical moving parts and electronics. Such structures can sense the variation of mechanical quantities or actuate. Most MEMS devices consist of a mass which is free to move in one or more directions in 3D space with respect to a substrate, to which it is anchored by springs. Different methods—capacitive, piezoresistive, optical, and resonant sensing—are commonly used to sense the displacement of the moving mass. Capacitive readout MEMS are based on the measure of a capacitance variation due to the displacement of a suspended microscopic structure in the presence of an external applied force. Moving electrodes (also called rotors borrowing mechanical terminology) are mechanically anchored to the moving structure and fixed electrodes (called stators as a consequence) are anchored to the substrate. Figure 1a shows a differential capacitive sensing cell with a moving electrode anchored to a suspended shuttle (on the right) and forming a couple of capacitors with stators A and B. A microelectromechanical system can be modelled as a lumped parameter spring-mass-damper system [11], as shown in Figure 1b: a mass is connected via a spring to a fixed support, being pulled by an external force *F_ext_*. A dashpot is used to represent a mechanical damping element. All these three elements share the same displacement *x* with respect to a rest position. For the sake of simplicity, only a 1-axis model is considered now, neglecting secondary vibrating modes; this analysis can be easily extended to a 3-DOF system in an inertial frame of reference. Applying Newton’s second law of motion, stating that the net force on a body is equal to the product of acceleration and mass of the body: *F* = *m × a*, the classical equation of motion describing the dynamics of a suspended micromachined structure can be derived:

(1) $$m\cdot \ddot{x}+b\cdot \dot{x}+k\cdot x=F_{ext}$$

being the elastic force proportional to the displacement x, the viscous force to the velocity $\dot{x}$. 

By applying Laplace transform to Equation (1), the frequency behavior of MEMS can be studied with respect to frequency and two main parameters can be highlighted to describe the behavior of the mechanical element. The resonance frequency is defined as:

(2) $$f_{r}=\frac{1}{2\pi }\cdot \sqrt{\frac{k}{m}}$$

A heavier moving mass resonates at lower frequency; the stiffer is the spring, the higher is the resonance frequency. 

The quality factor *Q* is a dimensionless parameter useful to characterize how over- or under-damped a MEMS resonator is. Equivalently, for large values, *Q* also characterizes resonator bandwidth Δf relative to its center frequency $f_{r}$ and it is related to MEMS parameters according to the expression:

(3) $$Q=\frac{f_{r}}{\Delta f}=\frac{\omega_{r}\cdot m}{b}=\frac{\sqrt{k\cdot m}}{b}$$

Micromechanical devices are affected by thermal noise like all dissipative systems. In particular, dimensional scaling is attractive for a higher density integration, but small moving parts become more susceptible to mechanical noise due to molecular movement. Especially in sensors targeted for very low signal applications, mechanical noise may be a limiting factor. The power spectral density of the noise force can be written as [12]:

(4) $$S_{Fn}=4k_{B}Tb$$

where T is the absolute temperature, $k_{B}$ the Boltzmann constant and b the previously introduced damping coefficient. It should not be surprising that the resulting expression for mechanical noise is very similar to that for Johnson noise in resistors, *SV*_n_ = 4*k_B_TR*, as they both have the same physical origin, dissipation.

In gas damped systems, like MEMS working either at ambient pressure or in a package at a lower pressure, mechanical noise is mainly due to the random paths of molecules which hit the suspended structure. The result of this statistic process is an unwanted random displacement of the moving mass which is nevertheless detected by the position sensing interface.

## 3. CDC with Self-Oscillated Noise-Shaping Integrating Dual Slope

**Figure 2:**
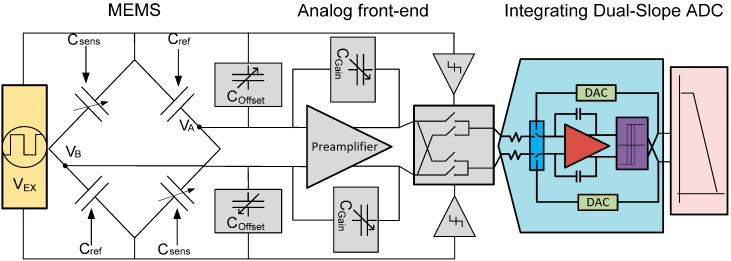
Block schematic of the MEMS sensor and CDC.

Taking in account the physical limitations introduced in Section 2, a capacitive MEMS and its readout circuit have been designed. The configuration of the CDC, using a MEMS with fully differential Wheatstone bridge, is shown in Figure 2.

This bridge has two different types of capacitive MEMS sensors. *C_sens_* is a micromachined sensing element whose capacitance has a major dependency on external pressure. This application is designed to work in a range of pressures from 800 hPa to 1300 hPa, and this pressure range corresponds to a capacitance range of a few pF in *C_sens_*. *C_ref_* is a reference capacitor, whose capacitance does not depend on pressure, but has the same temperature variation as *C_sens_*. The bridge is differentially modulated by a smoothed (to avoid higher order resonances that will affect the resolution) square wave. The output of the bridge is a square signal with amplitude proportional to the difference in capacitance of the two capacitor types. Equation (5) shows this relationship:

(5) $$V_{diff}=V_{A}-V_{B}=\frac{C_{sens}}{\left(C_{sens}+C_{ref}\right)}\cdot V_{EX}-\frac{C_{ref}}{\left(C_{sens}+C_{ref}\right)}\cdot V_{EX}=\frac{C_{sens}-C_{ref}}{\left(C_{sens}+C_{ref}\right)}\cdot V_{EX}$$

### 3.1. Front-End Circuit

Using this technique of modulation in the bridge, the low frequency signal information is transposed to the odd harmonic frequencies of the modulation signal [12]. The modulated signal is then processed by a voltage amplifier (Preamplifier in Figure 2) to perform a capacitive to voltage conversion. Additionally, the Preamplifier is intended to adjust different sensor capacitive ranges to the ADC input full-scale voltage. *C_offset_* and *C_Gain_* are programmable capacitors which are used to amplify or attenuate the bridge signal and to compensate for bridge offset at rest (i.e., unbalancing due to parasitic noises). With this feature, the solution is able to deal with minor variations of performance between different samples of the same type of MEMS or be readjusted to make the conversion of complete different application that use the same type of excitation in the MEMS. In order to obtain the DC information of the modulated and amplified signal of the bridge, a demodulation stage after the Preamplifier is needed. This stage is driven by a square signal (Figure 2), generated using the excitation signal through a buffer. The phase shift between the modulation and demodulation signal should be aligned to the phase shift of the amplifier stage in order to keep the DC gain of the amplifier stage at a maximum. 

**Figure 3:**
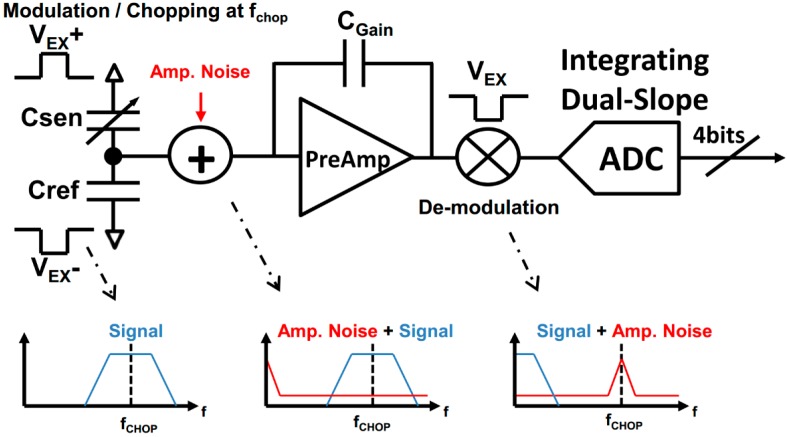
Modulation and chopping scheme.

It is important to mention that offset and low frequency (i.e., flicker) noise introduced by the Preamplifier is modulated only once by the demodulation stage and is therefore transposed to odd harmonics of the demodulation frequency (equal to the excitation frequency of the MEMS), ideally leaving the demodulated signal without any offset and low-frequency noise. This effect is illustrated in Figure 3. This procedure is referred to as chopping [12]. The frequency that modulates the MEMS (chopping frequency) needs to be selected in a way that the noise does not go into the bandwidth of the signal. However, it should be as low as possible to avoid multiple issues like: power consumption, DC Gain of the Preamplifier and ringing behavior in the output signal of the capacitive bridge. Note, that the Preamplifier is not chopped separately, but rather intrinsically chopped by the demodulation stage in the signal chain.

In this way the input of the DS ADC only digitizes the difference in voltage between the *C_sen_* and *C_ref_*, amplified by the Preamplifier. The ideal relation between bridge capacitors and DS ADC input voltage *V_IN_* (see Figure 3), is shown in (6):

(6) $$V_{IN}=-V_{EX}\cdot \frac{C_{sen}-C_{ref}}{C_{Gain}}$$

### 3.2. Self-Oscillated Noise-Shaping Integrating Dual-Slope ADC

**Figure 4:**
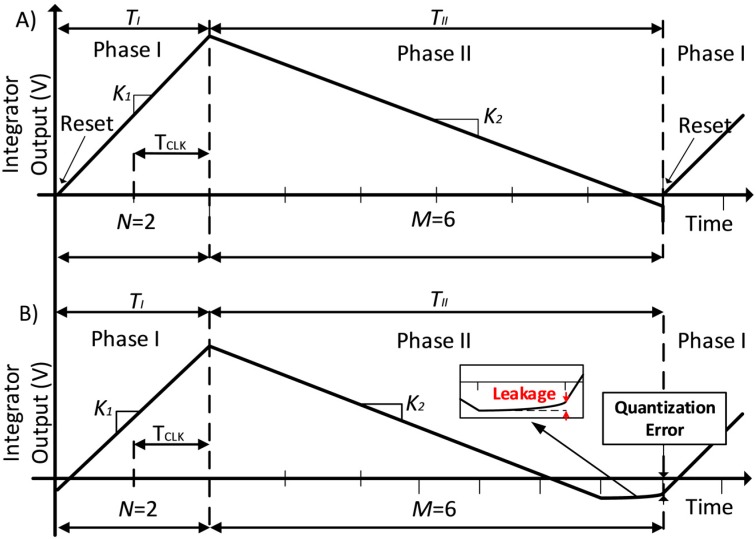
(**A**) Standard Dual Slope; (**B**) Standard noise-shaping Integrating Dual-Slope.

As mentioned in the Introduction, DS converters are good candidates for measuring low bandwidth signals with low power, simple architecture and high resolution. The DS architecture can be seen in the right part of Figure 3. The DS principles consists of charging first a capacitor in an active integrator with current proportional to the input voltage and after a fixed time, discharging the voltage stored in the capacitor with a fixed current until it crosses zero. The behavior can be seen in Figure 4A. The conversion starts in Phase I. The capacitor is connected to the input (output of the front-end circuit) for a fixed amount of time (*T_I_*). At the end of Phase I, the capacitor will have a voltage proportional to the output of the demodulator stage. Note that this voltage is a representation of the difference in capacitance of the sensor bridge. In the second step of conversion (Phase II), the capacitor is connected to a reference voltage using a 1-bit DAC (green blocks in Figure 2). The capacitor will be discharged by a fixed amount of current until the output of the integrator crosses zero. The amount of clock cycles (*M*) in Phase II will determine the digital output obtained in each conversion time (the maximum digital output number would be 6 in the example of Figure 4A). After this, the capacitor would be reset and the next conversion cycle will start. However, this classic approach [13] has big limitations and it is not efficient enough to compare with other techniques. The main reason is the time cost of achieving high resolution. The resolution (*N_bits_*) is proportional to the number of clock cycles needed to discharge the voltage (7). In order to increase the resolution, parameter *M_MAX_* will increase exponentially. This would lead to a higher clock rate and power consumption:

(7) $$N_{bits}=\log_{2}\left(M_{MAX}\right)$$

To improve the performance of classical DS converters, the topology called noise-shaping integrating DS was presented in [8,9,10]. In these works it was shown that by modifying the traditional dual-slope ADC the latency can be reduced. This is done by a slight modification of the traditional architecture. This modification is based on the fact that the capacitor voltage at the end of each sampling period is not reset (as a difference with traditional dual-slope ADC where this capacitor voltage is reset). This is shown in Figure 4B. This difference implies that the quantification error is kept for the next sampling period. This way first order noise shaping and latency reduction is also achieved. However, to do so, it is needed to have a fixed sampling time (*Ts* = *T_I_* + *T_II_*). For this, a slight modification of the DS ADC equations is needed. In this case, the variables of the converter, must be selected in a way that the system is able to discharge completely the integrating capacitor *C_in_* in Phase II when the input signal is at its maximum value. The control of the discharge in Phase II depends on the following two equations:

(8) $$K_{1}\;\cdot \;N\;\cdot \;T_{clk}+V_{LSB}=K_{2}\;\cdot \;M\;\cdot \;T_{clk}$$

(9) $$K_{2}=\frac{I_{feedback}}{C_{int}}$$

In addition, some circuit design related issues must be still solved. First, the integrating capacitor must keep its charge constant after the zero crossing until the next sampling period. Parasitic of the circuit plays an important role in this case and takes a significant unwanted amount of charge from the capacitor (leakage). This leads to a value of the quantization error that, at the end of the sampling period, is different from the estimated one after the zero crossing. Second, the transfer function is not linear. The possible output digital values are in the range from −M to M. However, there are two different “0”, “0+” and “0−”. To compensate this, an extra digital logic is needed (two different approaches were already shown in [7,9]), increasing the area and power consumption of the converter.

**Figure 5:**
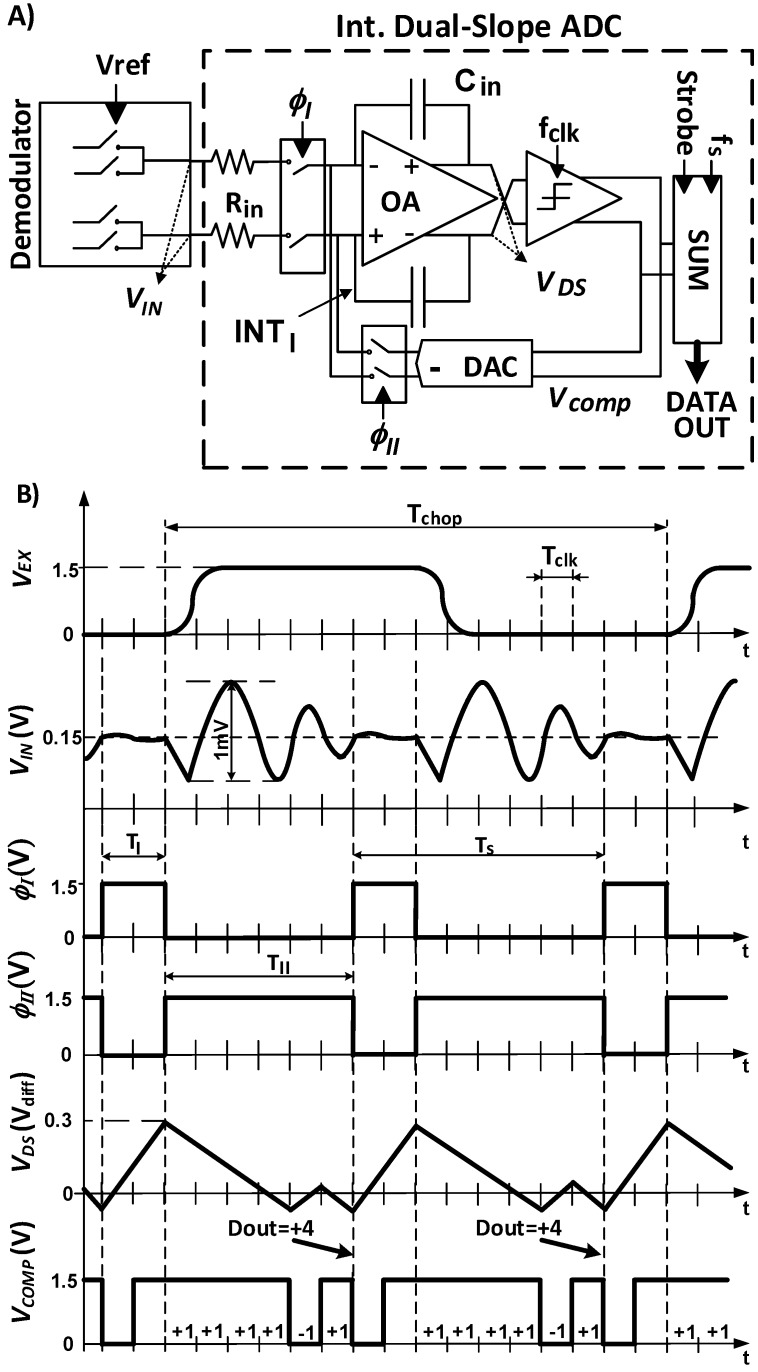
(**A**) Self-Oscillated noise-shaping Integrating Dual-Slope scheme; (**B**) Time diagram of the CDC.

In this work a new timing scheme is proposed in order to simplify the extra digital logic needed in the previous topology and reduce the influence of the leakage in the quantization error value. Figure 5A shows the proposed architecture and Figure 5B its time diagram. The main difference with the standard noise-shaping integrating DS (Figure 4B) is that once the voltage of the integrator crosses zero, the quantization error value is not stored in the integrating capacitor for the next sample. Instead, the DAC keeps toggling until the next sampling period. In [7] it was demonstrated that by adopting this modification the final value of the voltage in the output of the integrator at the end of the sampling period (end of phase II; Ф_II_ in Figure 5B) still represents the quantization error. This way, the quantification error is noise-shaped, the same way as in the architecture of Figure 4B. However this configuration does not need an extra digital control circuit for the auto-zero compensation, because the self-oscillating behavior already performs this compensation. Furthermore, now leakage is not an issue as the quantization error does not need to be stored in any capacitor. These modifications save area and power compare with previous solutions. 

This new topology is known as “self-oscillated noise-shaping integrating dual-slope converter”. As it was mentioned before, the readout circuit is modulating the input signal to high frequencies to remove offset and flicker noise in first part of the chain. In Figure 5B *V_EX_* represents the excitation signal of the MEMS bridge. It is shaped in a pseudo-trapezoidal wave in order to reduce stimulation of high frequency of MEMS sensor and ringing in the output voltage of the bridge. The excitation of the MEMS together with the demodulation block makes the chopping of the first part of the CDC. As it was mentioned before, the chopping frequency must be carefully chosen: it must be high enough to modulate flicker above bandwidth of interest; but low enough to allow the signal stabilizes after the ringing. The output of the demodulator is the input of the Self-Oscillated noise-shaping Integrating DS converter (*V_IN_* in Figure 5A). As it was explained, the signal is affected by the ringing of the MEMS. Taking into account the desired frequency of the chopping, the pseudo-trapezoidal waveform is designed in a way that the signal will have a stable value for a specified length of time every chopping semi-period.

This specified length of time will be assigned for the Phase I of the self-oscillated noise-shaping integrating DS converter (as a track phase in a track and hold stage). If the length of Phase I is larger than the length of time where *V_IN_* is stable, the ripple of the output of the Preamplifier will affect the output of the CDC reducing the resolution. The signals Ф_I_ and Ф_II_ represent the two phases of the self-oscillated noise-shaping integrating DS period. In order to achieve high resolution with the lowest clock frequencies (to reduce power consumption), the two phases of the conversion and therefore, N and *M*, are selected unequal. If Phase II is larger, *M* will be larger and there for the number of bit inside the dual-slope quantizer. N is equal to two clock periods, which is the minimum value for Phase I for a proper behavior of the system, giving enough time to integrate the input signal. The clock frequencies are selected in a way that this amount of time (*N*·*T_clk_*) is equal to the stable time of *V_IN_*. *M* is equal to 6. As it was mentioned before, *V_DS_* (Figure 5A) is the output of the integrator of the Self-Oscillated noise-shaping Integrating DS (red block in Figure 2). Signal *V_COMP_* represents the output of the clocked comparator (purple block in Figure 2). This signal will be used to drive the DAC and to generate the multibit digital output of the CDC through the digital filter (pink block in Figure 2). This block makes the logic addition of the output digital data only during Phase II (high level adds a 1 and low level subtracts a 1) every falling edge of Phase II (λ_2_). Digital output is then proportional to the input amplitude of the DS converter (*V_IN_*), and therefore to the input pressure of the CDC. In this system, the sampling period is defined as *Ts* = *T_I_* + *T_II_*, where *T_I_* = *N*·*T_clk_*, *T_II_* = *M·T_clk_*. and *T_clk_* is the clock period of the comparator.

**Figure 6:**
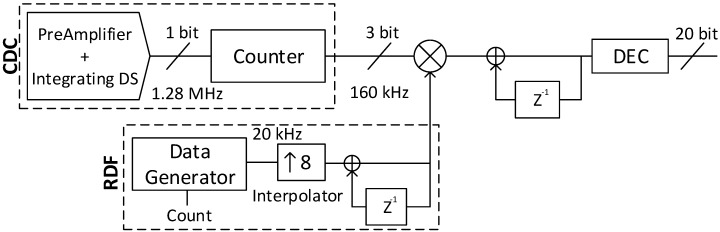
Block schematic of the reconfigurable digital filter.

In order to measure DC input values with high resolution a reconfigurable digital filter (RDF) has been used. For this prototype, the RDF has been implemented in MATLAB. A diagram block of this filter is shown on Figure 6. The Data Generator can be configured for the resolution that is needed, using the Counter. The resolution depends on the measuring time, which is F_L_·Ts, where F_L_ is the length of the filter. The data of this block is generated with low frequency (20 kHz) in order to save power. After the Data Generator, an interpolator is used to increase the frequency of this data to the sampling frequency. After integrating the data, the *tukey* window is created. The output of the RDF multiplies the output of the ADC. The result goes through a configurable low pass filter to obtain a digital number adjusted to the resolution required. In our experiments, the configuration will be for high resolution, the RDF will be configured to give 20 bits.

### 3.3. Circuit Design

The analog front end is based on a closed loop capacitive voltage amplifier (Preamplifier in Figure 2) built with a telescopic gain-boosted OTA in order to be power efficient. This amplifier does not need any specific technique to reduce low frequency noise as it is already chopped by the modulated/demodulated scheme proposed in this architecture.

The power consumption of the DS converter is given by the RC integrator (INT_I_ in Figure 5A). The OTA used in the integrator is a two stage class A/AB pull-up-down topology and it is Miller-compensated. This architecture allows a better power tradeoff with respect to folded cascode and two stage class A topologies. Moreover, to cope with the 1/*f* noise introduced by the OTA input differential pair a chopping modulation technique has been adopted. The signal demodulation is applied at low impedance nodes. This chopping configuration does not suffer from the OTA limited bandwidth and from the distortion introduced by the chopping switches. This is because the demodulation of the chopping stage is performed before the dominant pole and not at the output of the OTA. The chopping frequency of the OTA is the same that does system chopping in the front-end circuit, this way it is aligned with the timing of the system. The DC gain is G_DC_ = 80 dB. The gain bandwidth product of this OTA has been set to 4 times the clock frequency GxBW = 4·*f_clk_* = 5 MHz (*f_clk_* = 1/*T_clk_*) in order to deal with the DAC pulses. The DAC uses a steering current topology. IDAC uses non-return-to-zero pulses with a current I_DAC_ = 1.5 µA. In order to deal with the 1/*f* noise at the output of the IDAC, the current mirrors that drive the current cells are designed with large size PMOS (W = 9 µm/L = 94 µm) and NMOS (W = 9 µm/L = 150 µm) transistors. A two-stage regenerative low power clocked comparator is used for the single-bit conversion. Its output is a PWM waveform that can be directly connected to the IDAC and to the counter SUM to generate D_OUT_. 

## 4. Measurements

**Figure 7:**
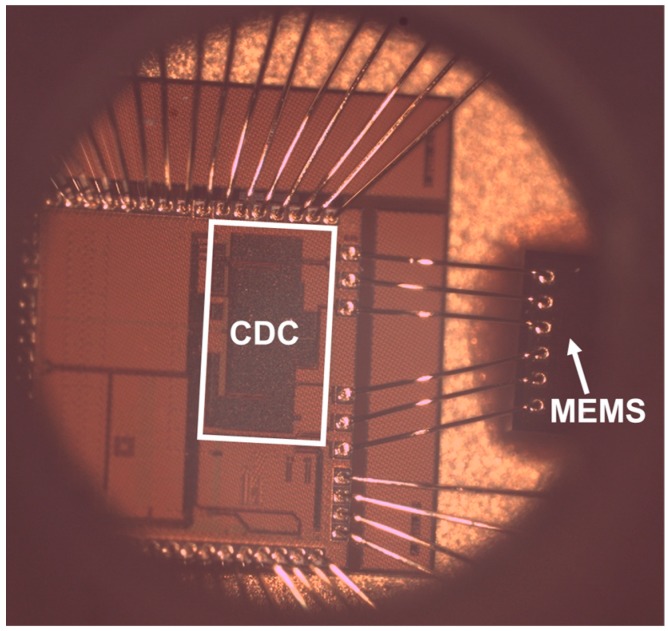
Die photo of the MEMS sensor and the CDC bonded together in a package with a hole for pressure control.

To obtain experimental results, the proposed CDC was fabricated in a standard digital 0.13 µm CMOS technology. The CDC was bonded together with a pressure sensor MEMS to minimizes the parasitic capacitances between them. The bonding is done in a ceramic carrier of 64 pins. The cover of the package has a hole on top to give the possibility of controlling the air pressure inside this cavity. Figure 7 show a die photo of the MEMS sensor and the CDC bonded together in a package with a hole for pressure control. 

The CDC core has an area of 0.317 mm^2^. The chip input master clock is set to 1.28 MHz, leading to a sampling frequency of 160 kHz. The excitation signal that drives the bridge of the capacitor MEMS, demodulates the output voltage of the Preamplifier and chops the DS integrator is set in this setup to 80 kHz. The chip consumes 146 µA when connected to a 1.5 V power supply. This current includes analog, digital and excitation signal generator blocks.

Each sample is welded to a small PCB that is connected into the socket of the mother PCB. This PCB is designed with discrete components and has the goal of control all the programmability of the digital inputs (offset and gain of the Preamplifier and different modes for test), do the readout of the output of the CDC and generate the necessary voltages for the different parts of the PCB.

**Figure 8:**
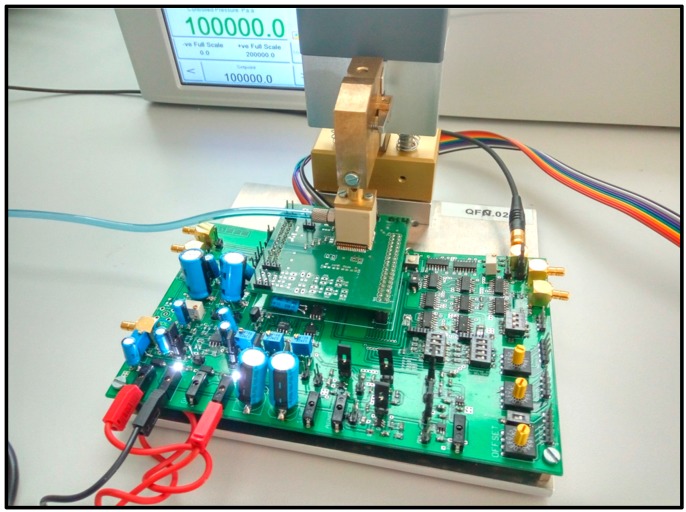
Test-chip welded and working connected to the test PCB and pressure controller.

The system connected and working can be seen in Figure 8. In this figure the vacuum cup that creates the isolated chamber for pressure testing is also connected. In this way, the pressure controller (also in Figure 8) can provide a stable value of pressure for the measurement (1000 hPa in this example).

Here is the list of hardware that has been used during the whole measuring process to make all the experiments:

- Oscilloscope: Tektronix DPO 5034 with BW = 350 MHz and 3 Gsamples/s.
- Clock generator: Tektronix AFG 310.2
- Power supply: Agilent E3631A Triple Output DC Power Supply.
- Pressure controller: Druck Pace 5000
- Bitstream Analyzer: GP_24132
- Temperature test chamber: Vötsch VT 7004.

The master supply voltage and the mother clock used in the PCB are ±5 V and 2.56 MHz respectively. The output data is captured using the official software of the Bitstream Analyzer. This software generates a text file that has a one bit stream with the outputs of the CDC. The file is processed later on with MATLAB.

**Figure 9:**
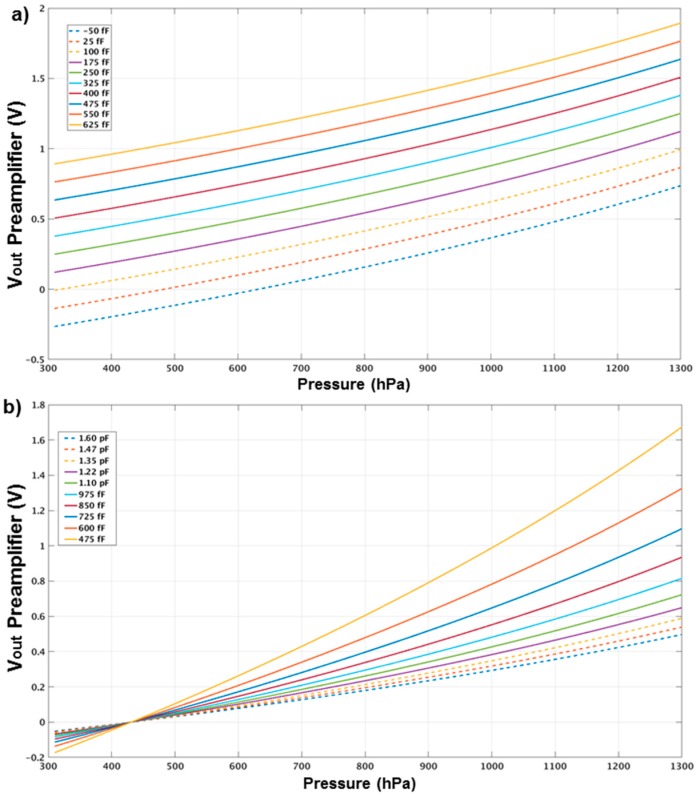
Use of configurable capacitors in offset and gain in the Preamplifier: (**a**) single ended outputs of the Preamplifier with range of offset capacitors; (**b**) single ended outputs of the Preamplifier with range of gain capacitors.

To start with the measurements the readout circuit needs to be configured. Figure 9 shows examples of transformation of the signal into the desire range thanks to the programmability of the CDC. This topology is able to give higher resolution if the zero and full-scale of sensor are aligned with the zero and full-scale of the DS ADC. The DS ADC in this prototype is designed to work with a voltage range of 1 V differential. As it was mentioned before in Section 3.1, the gain and offset capacitors of the Preamplifier can be tuned according to the input range of the sensor to reach the full-scale of the ADC. The proper adjustment of these values can be seen in Figure 9a (offset capacitors) and Figure 9b (gain capacitors). 

**Figure 10:**
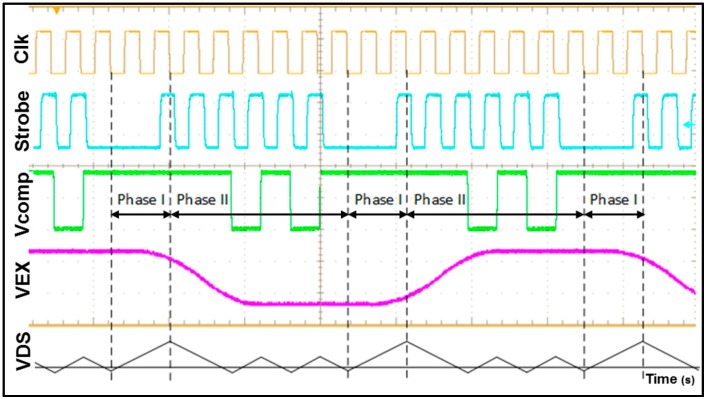
Time diagram of the signals that proves the behavior of the noise-shaping Integrating Dual-Slope measured from the test-chip.

To check that the CDC is behaving as expected, some selected signals from the chip are captured using an oscilloscope. Figure 10 shows these signals that are similar to the ones explained in Section 3.2 (Figure 5). 

It can be seen that both figures (Figure 5 and Figure 10) show the same behavior, as expected. “Clk” is the master clock of 1.28 MHz used to create all the phases and lower clocks. Strobe is the signal created to capture data from the comparator output. It can be seen that the data is only captured in Phase II. *V_EX_* is the signal that drives the MEMS bridge. *V_COMP_* is the digital signal obtained after the comparator and the one that is saved every rising edge of Strobe. VDS is added to make easier to understand how the system is working compare with Figure 5.

**Figure 11:**
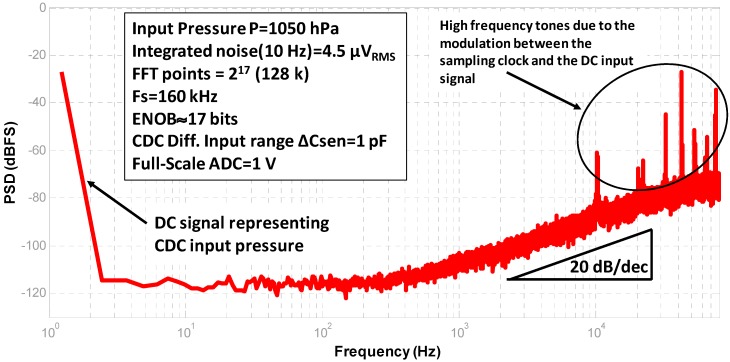
FFT of the CDC for an input pressure of 1050 hPa.

Ten different samples have been measured to study and verify the robustness of the design. To measure these samples the pressure controller was attached to the top of the package. A constant pressure with an error of ±0.1 Pa is set by the Druck Pace 5000. The spectrum of the digital output of the CDC under these conditions is shown in Figure 11. It represents an input pressure of 1050 hPa, equivalent to a −16 dBFS at the input of the DS ADC, where the Full-Scale is VFS = 1 V. The measured equivalent integrated noise over a bandwidth of 10 Hz is 4.5 µ*V_rms_*. Using Equation (8), a maximum signal-to-noise-ratio (SNR_max_) of 103.9 dB is obtained. This is equivalent to an effective number of bits (ENOB) of 17 bits. Also, in Figure 11 the first order noise shaping from the self-oscillated noise-shaping Integrating DS converter can be observed. The modulation between the DC signal and the clock is present as well. This behavior is well known in first order noise shaping ADCs. The tones that can be seen at high frequencies do not affect the in-band noise floor, and therefore, the resolution:

(10) SNRmax=20∗log10(VFS2Vrms)

**Figure 12:**
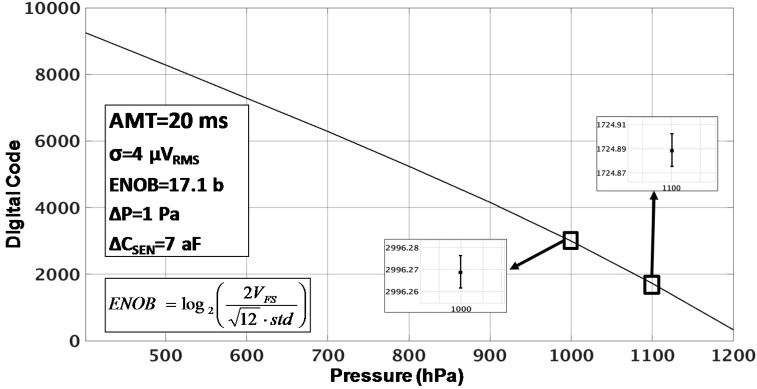
Digital output code of the CDC vs. input pressure in differential measurements analysis.

The purpose of this implementation is to achieve high resolution and linearity for relative measurements (not absolute pressure measurements). To achieve this, the resolution of the CDC can be measured by another approach. The pressure is swept from 400 hPa to 1200 hPa using a step of 25 hPa. The output of this measurement can be seen in Figure 12. Then, at each pressure point, 100 samples have been captured using a measuring time of 20 ms each. It can be proved that this time can be modified in order to tradeoff measuring time by resolution. After, using a sync filter, a standard deviation of σ_rms_ = 4 µ*V_rms_* is obtained for each pressure point. This standard deviation leads to a resolution of ΔC = 7 aF or ΔP = 1 Pa, which is equivalent to an ENOB of 17.1 bits (this value has been calculated using the equivalent formula of Equation (10) shown in Figure 12. This result is coherent with the noise spectral density shown in the FFT of Figure 11.

**Figure 13:**
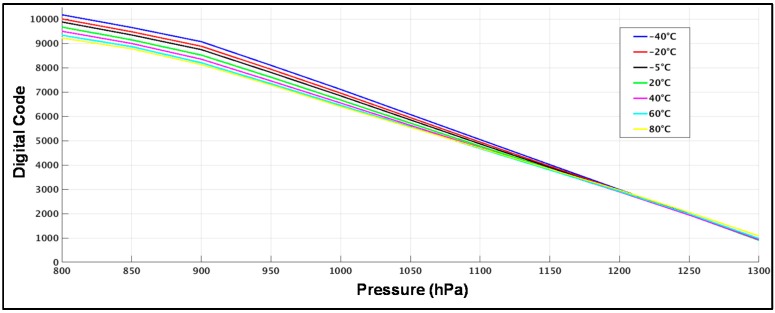
RAW data out of the transfer characteristics measured in the prototype for different temperatures.

Taking into account that the direct application of the CDC is for different sensors and environments, a final experiment has been developed to check the effect of temperature variations on performance. For this experiment the prototype has been placed inside a temperature test chamber. A range from −40 to 80 °C has been covered. To have a better control of the temperature in the chip a PTC 100 of four channels is attached close to the package. In this way a resolution of ±0.5 °C is provided. For each temperature, the same kind of measurements used in the previous case has been done. The output of this measurement is a transfer function for each temperature point. This result is shown in Figure 13. The data presented is RAW data. No compensation or trimming has been done.

**Figure 14:**
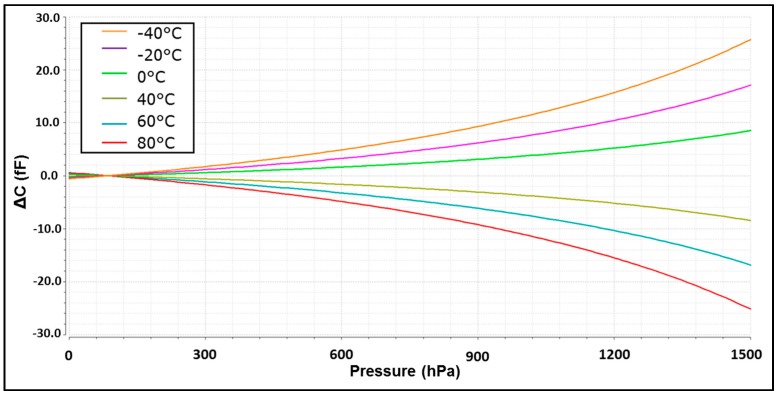
Error of resolution in the MEMS depending on the pressure for each temperature.

**Figure 15:**
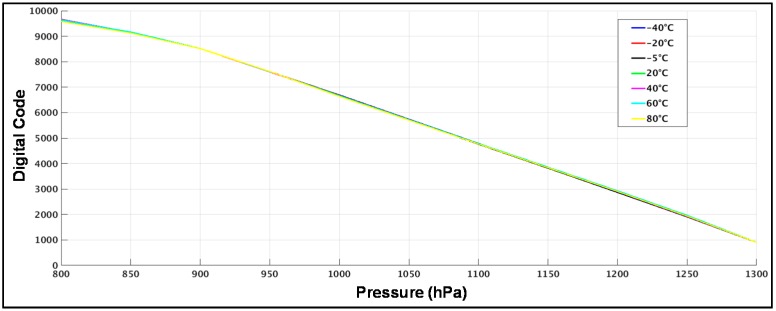
Compensated transfer characteristics after temperature, offset and gain error for different temperatures.

Trying to merge all the transfer characteristics to a single one is important to take into account all the factors that contribute in each curve that are under control. The first one is the error related to the temperature dependency of the MEMS. The ΔC between the *C_sens_* and *C_ref_* is not only dependent on pressure. Different temperatures introduce a different offset in the ΔC for each pressure point as it can be seen in Figure 14. Using for each temperature the curves presented in this figure, the correction factor is applied digitally. In our experiments, this correction was done directly in MATLAB. Once the temperature error is solved, a correction of offset and gain is applied. For this design a polynomial of order one (*y* = a*x* + b) is used. The final output after all the correction factors is presented in Figure 15. The resolution between different curves is, ENOB(T) = 11 bits. This number means an error resolution of ΔC = 44.5 aF and ΔP ≈ 7 Pa between different temperatures. It is important to take in account that this value represents absolute resolution. It is not the same as the differential resolution explained before.

## 5. Discussion and Conclusions

**Figure 16:**
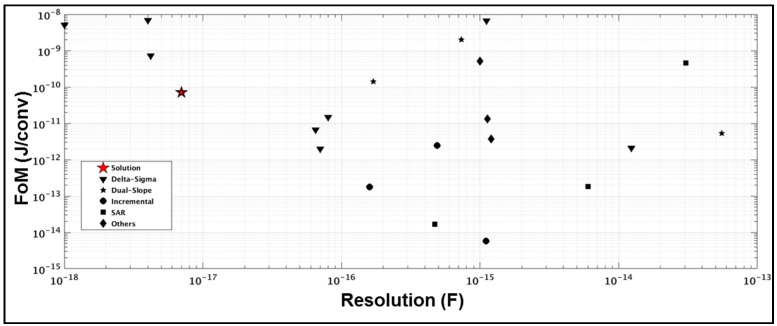
State-of-the-art plot. Figure of merit (FoM) vs. resolution.

After proving the performance of the CDC, a comparison with other state-of-the-art solutions is presented. Due to the significantly increasing Internet of Things (IoT) market in the last years, different approaches are being used to cope with the trade-off between performance and low power consumption: Sigma-Delta modulation, SAR, incremental ADCs and dual-slope converters are the main topologies. Sigma-Delta modulators are well suited to achieve higher resolution than other topologies. However, they have to deal with bigger area and power demanding blocks. On the other hand, SAR converters are very efficient in power consumption but they are not able to achieve as much resolution as Sigma-Delta modulators. Incremental converters are becoming more popular nowadays. They are able to achieve high resolution with some advantages compared with Sigma-Delta modulators: a simpler decimation filter, ease of multiplexing, low latency, and the absence of idle tones. However, the circuit complexity of these converters is high. The matching between stages needs to be perfectly done in order to keep performance. This feature makes this kind of converters weak against process, voltage and temperature (PVT) variations. In this work, an incipient new family of converters is presented. The proposed self-oscillated noise-shaping integrating dual-slope device is able to achieve the same performance as incremental converters, using the same range of power consumption, but with a simpler configuration. This benefit makes this solution stronger against PVT variations as it was shown in Section 4. The solution includes an analog front end and uses a simple decimation filter. Also, due the digital control, it is able to multiplex different inputs and change the resolution of the converter in an efficient way. Figure 16 shows a plot with the state-of-the-art converters and this work using the data of Table 1. This table shows that the topology presented in this work has similar SNR and FoM as the state-of-the-art CDCs for the same capacitance resolution. As mentioned before Sigma-Delta modulators are able to achieve higher resolution at the expense of higher power consumption. SARs converters are power efficient, but they are not able to achieve high resolution. Incremental converters are also power efficient and they can achieve higher resolution, but as mentioned before, their behavior against PVT makes them a poor candidate for CDC products. Finally classical DS are not efficient enough for these applications.

In summary, it can be seen in Figure 16 that there is a tradeoff between FoM and resolution in all the converters. All of them use different measuring times or different power consumption to achieve different resolution, but somehow the change of these variables is just moving the solutions over the red line. In this scenario, the proposed CDC offers a power efficient solution using very simple and robust implementation. In addition, its digital control gives an easy scalability and because of that, the solution can be more flexible than the competitors in order to work for different sensors using the same hardware.

This paper shows experimental results of a high resolution CDC based on a self-oscillated noise-shaping integrating DS converter that can work for different types of MEMS sensors. It achieves a capacitance resolution of 7 aF that is equivalent to a resolution of 1 Pa. It is a competitive solution compare with the state-of-the-art without using multi-bit circuits. Instead of that it uses time domain circuitry to exchange amplitude by time resolution. In addition, this solution has a more efficient tradeoff between measurement time, power and resolution compared with other CDCs. With the results it is proven that the solution has small sensitivity to temperature and process variations, simplicity to modify offset or gain parameters to correct the sensor spread and an area and energy efficient implementation. For all these reasons, the presented CDC seems to be a good candidate for sensor readout circuits in IoT.

## Figures and Tables

**Table 1:** CDC state-of-the-art.

Reference	Type	Measure Time (ms)	Power (µW)	Sensor Range (pF)	Resolution Cap. (aF)	SNR_cap_^1^ (dB)	FoM ^2^ (pJ/conv)
[2]	ΔΣ	0.020	15000	10	65	94.71	6.75
[3]	Inc.	0.23	33.7	24	160	94.5	0.179
[4]	ΔΣ	0.8	10	1	70	74	2
[5]	ΔΣ	0.019	1.84	0.7	12,300	26.1	2.13
[6]	DS	7.6	211	6.8	170	83	139
[13]	DS	6.4	0.1	25.4	55,300	44.2	5.31
[14]	ΔΣ	1090	3750	8	4.2	116.6	742
[15]	ΔΣ	100	60000	4	1	123	5190
[16]	ΔΣ	13.3	6000	0.16	4	83	6,900
[17]	ΔΣ	10.2	10	2	80	78,9	14.9
[18]	ΔΣ	100	7	0.4	1110	42.1	6730
[19]	Inc.	0.001	1440	1	490	57.2	2.44
[20]	Inc.	0.001	7.5	5	1100	64.1	0.006
[21]	SAR	4	0.16	72.8	60	72.6	0.183
[22]	SAR	0.005	6.7	3.2	470	67.6	0.017
[23]	SAR	100	0.8	18.5	30,400	46.7	455
[24]	Digital	1	0.27	0.3	1200	38.9	3.74
[25]	Current	0.004	725	0.75	1130	47.4	13.2
[26]	DS	0.02	15800	0.4	733	45.7	2010
[27]	Current	0.002	220	1.8	800	58	0.67
This work	Int. DS	20	220	1	5.4	96.3	82.2

^1^ $SNRcap(dB)=20\log\cdot \left(\frac{In.Range/2\sqrt{2}}{resolution}\right)$; ^2^ $FoM=\frac{Power\times Meas.Time}{2^{(SNRcap-1.76)/6.02}}$.
